# PDGF-BB Promotes Type I IFN-Dependent Vascular Alterations and Monocyte Recruitment in a Model of Dermal Fibrosis

**DOI:** 10.1371/journal.pone.0162758

**Published:** 2016-09-12

**Authors:** John S. Cho, Terry C. Fang, Taylor L. Reynolds, Daniel J. Sofia, Stefan Hamann, Linda C. Burkly

**Affiliations:** 1 Immunology Research, Biogen, Cambridge, MA, United States of America; 2 Translational Sciences - Pathology, Biogen, Cambridge, MA, United States of America; University of Texas Health Science Center at Houston, UNITED STATES

## Abstract

Systemic sclerosis (SSc) is a chronic autoimmune disorder that can result in extensive tissue damage in the skin and, in advanced cases, internal organs. Vasculopathy, aberrant immune activation, and tissue fibrosis are three hallmarks of the disease that have been identified, with vasculopathy and aberrant immunity being amongst the earliest events. However, a mechanistic link between these processes has not been established. Here, we have identified a novel role of platelet derived growth factor-BB (PDGF-BB)/PDGFRβ activation in combination with dermal injury induced by bleomycin as a driver of early, aberrant expression of interferon stimulatory genes (ISGs) and inflammatory monocyte infiltration. Activation of PDGFRβ in combination with bleomycin-induced dermal injury resulted in increased dermal thickness, vascular density, monocyte/macrophage infiltration, and exacerbation of tissue injury. Many of these features were dependent on IFNAR-signaling, and an increase in the number of interferon-beta (IFN-β) producing monocytes cells was found in the skin lesions. Taken together, these results identify a novel link between PDGFRβ activation, and Type I IFN-driven vascular maintenance and monocyte/macrophage cell recruitment, and provide a potential explanation linking key features of SSc that were previously thought to be unrelated.

## Introduction

Systemic sclerosis (SSc) is a complex multi-system autoimmune disease with three distinct pathologies that define the disease, namely: vasculopathy, aberrant immune activation, and tissue fibrosis [1]. While vasculopathy and aberrant immune activation occur in early stages of SSc, the advanced stages are marked by tissue fibrosis that develops in the skin and can progress to other organs, leading to mortality. TGFβ has been implicated as a driving factor for differentiation of pathogenic collagen-producing myofibroblasts which are responsible for the excessive deposition of extracellular matrix proteins seen in advanced SSc [2]. However, the upstream vascular and immune events leading to fibrosis and their influence on other aspects of SSc disease pathogenesis has not been established.

One of the earliest clinical manifestations of SSc is Raynaud’s phenomenon, a vasculopathy characterized by an excessive vasospasm response to cold or stress, which can precede the diagnosis of SSc by months or even years. Microvascular alterations and loss of small vessel function can ultimately lead to ischemia in peripheral tissue leading to digital ulcers or more severe complications such as pulmonary arterial hypertension. In general, the vascular manifestations of SSc primarily affect the structure and function of small vessels, resulting in abnormally structured nail-fold capillaries, telangiectasia, and loss of capillaries [3]. Interestingly, despite the apparent loss of small blood vessels, pro-angiogenic factors, such as PDGF-BB and VEGF, are highly expressed in SSc skin lesions for reasons unknown [4,5].

PDGF-BB is a multifunctional growth factor with mitogenic activity on cells of the mesenchymal lineage including fibroblasts and perivascular pericytes [6]. In SSc, PDGF-BB and its receptor, PDGFRβ, are upregulated in lesional skin biopsies [4,7]. PDGFRβ expression appears to be particularly enhanced on pericytes surrounding the dermal microvasculature in patients with early SSc, suggesting that PDGFRβ activation may have a role in modulating vascular function [8]. Further, PDGF receptor activating auto-antibodies have been reported in SSc patients [9]. Taken together, these observations suggest that altered activation of the PDGF-BB/PDGFRβ-axis may play a role in SSc disease pathogenesis.

In addition to the PDGF-BB/PDGFRβ-axis, Type I interferons have also been implicated in the pathogenesis of SSc in various studies. In a study of peripheral blood mononuclear cells (PBMCs) and skin lesions of SSc patients, an increased expression of Type I IFN-associated genes was observed compared with controls [10,11]. A separate study showed that approximately half of SSc patients have increased expression of an “interferon signature” [12]. Further supporting the role of Type I IFNs in SSc, the development of SSc-like skin manifestations was reported in patients being treated with interferons for the treatment of viral infections [13,14]. Interestingly, patients with genetic mutations leading to the overproduction of Type I IFNs (termed STING-associated vasculopathy with onset in infancy (SAVI) patients) have been reported to have systemic inflammatory responses with evidence of cutaneous vasculopathy and pulmonary inflammation [15]. Many of the clinical manifestation observed in SAVI patients echo those observed in SSc patients, including Raynaud’s phenomenon, telangiectasia, abnormal nail-fold capillaries, and digital ulcers [15].

While vasculopathy and immune cell activation in SSc are thought to be distinct features of the disease, there is some evidence that these processes may in fact be functionally linked. For example, vascular inflammation in SSc is evidenced by the presence of perivascular immune cell infiltrates, predominantly of the monocyte/macrophage cell lineage, in the affected lesions of SSc patients [16]. Further, mice engineered to express a constitutively active form of PDGFRβ exhibit increased pericyte coverage in capillaries with evidence of aberrant production of interferon stimulatory genes (ISGs) and other inflammatory mediators by brain vascular cells [17]. These mice do not develop tissue fibrosis, but they do exhibit extensive signs of perivascular inflammation and increased accumulation of myeloid cells in peripheral tissues. To investigate the roles of PDGF-BB and Type I IFNs in vasculopathy and aberrant immune cell activation in SSc, we developed a novel experimental model, which features PDGF-BB-dependent vascular activation, in combination with bleomycin-induced dermal injury and fibrosis. We used this model to study the complex interactions that occur at the site of disease, and identify the mechanisms by which these interactions may influence SSc disease pathogenesis.

## Materials and Methods

### Animals

C57BL/6J and IFNAR-deficient mice were obtained from The Jackson Laboratory (Bar Harbor, ME: Stock number: 000664 and 010830, respectively).

### Ethics statement

All animal studies were approved by the Institutional Animal Care and Use Committee (IACUC) of Biogen (Protocol #516) and performed in accordance with the Guide to the Care and Use of Laboratory Animals.

### Reagents

Recombinant murine PDGF-BB was purchased from Genscript (Piscataway, NJ) and pharmaceutical-grade bleomycin was purchased from Teva Pharmaceuticals (Cambridge, MA). All reagents were reconstituted in sterile saline.

### Experimental animal model of cutaneous fibrosis

Adult female C57BL/6J or IFNAR-deficient mice (approximately 8–10 weeks of age) were used in all experiments. One hundred μl of saline, PDGF-BB (200 ng), bleomycin (0.05 units), or a PDGF-BB (200 ng) + bleomycin (0.05 units) mixed solution was injected intradermally (using a 27-gauge needle) into a single location on the back, daily for 21 days. On days 3 and 21 after the first injection, mice were sacrificed, and skin at the site of injection was harvested for histology or RNA extraction as described below.

### Histology

Lesional 8-mm punch biopsy skin specimens were obtained on day 21 after treatment, fixed in formalin, and embedded in paraffin. Immunohistochemistry (IHC) was performed on 5 μm sections of formalin-fixed paraffin embedded tissue using a Discover XT automated stainer (Ventana Medical Systems). H&E, Masson's trichrome, and Picrosirius Red (PSR) staining were also performed according to standard methods. For CD31, IBA-1 (ionized calcium-binding adapter molecule 1), and PDGFRβ IHC staining, tissues were incubated with primary anti-CD31 (rabbit monoclonal, clone D8V9E; Cell Signaling; 0.04 μg/ml), anti-IBA-1 (rabbit polyclonal; Wako; 1 μg/ml), or anti-PDGFRβ (rabbit polyclonal; Sigma; 20 μg/ml). Tissue sections were counterstained with hematoxylin and visualized by light microscopy.

### Confocal microscopy

For PDGFRβ/CD31 dual immunofluorescence staining, antigen retrieval was performed with Tris/Borate/EDTA buffer, pH 8.0–8.5 (Ventana) for 1 hour at RT. Tissue sections were then incubated with primary anti-PDGFRβ (rabbit monoclonal, clone 28E1; Cell Signaling; 40 μg/ml) and anti-CD31 (rat monoclonal, clone SZ31; Dianova; 0.3 μg/ml) for 1 hour at 37°C. Secondary antibody staining was performed sequentially for the two antigens. First, tissue sections were stained with an OmniMap anti-rabbit HRP and developed with Discovery FITC substrate, followed by a peroxidase inhibitor step to quench residual HRP activity. Next, tissue sections were stained with an OmniMap anti-rat HRP and developed with Discovery Rhodamine substrate. Tissue sections were counterstained with the nuclear DNA stain DAPI and visualized by confocal microscopy.

### Image analysis

Quantification of dermal thickness, relative collagen, CD31, and IBA-1 immunopositive area were performed using customized algorithms on Visiopharm image analysis software (Hoersholm, Denmark). Dermal thickness was determined based on the Trichrome-stained samples, by using an H&E Hematoxylin filter to identify the dermal area. Ulcerated area was determined using manual annotation to delineate the perimeter. Total collagen was determined based on Picrosirius Red- stained samples using a Chromaticity Red filter to calculate relative area in the dermal and hypodermal area of the whole slide image. CD31 and IBA-1 positive areas were identified using a DAB filter applied to calculate relative area in the dermal and hypodermal area of the whole slide image. With the exception of dermal thickness, which only included dermis, all parameters were quantified down to the level of the panniculus carnosus.

### Flow cytometric analysis of skin single-cell suspensions

Mice were treated with saline, PDGF-BB, bleomycin, or PDGF-BB + bleomycin, as described above. Twenty-four hours after the third injection, mice were euthanized and 12-mm skin biopsies were collected and processed into single-cell suspensions as previously described [18]. Briefly, excess fat first removed from the underside of the skin biopsy and the remaining tissue was cut into small pieces. Skin tissue was digested in RPMI 1640 supplemented with 10 mM HEPES, 2.6 units/ml Liberase TM (Roche), 100 μg/ml DNase I (Roche), 0.5 mg/ml Hyaluronidase (Sigma), and 1X Penicillin/Streptomycin solution (Invitrogen) for 1 hour at 37°C with gentle agitation. After enzymatic digestion, the cell suspensions were placed in gentleMACS C tubes (Miltenyi) and further disrupted using a gentleMACS homogenizer (Miltenyi) before filtration using a 100 μm MACS SmartStrainer (Miltenyi) for FACS staining. Following a blocking step with FcBlock (BD Biosciences), cell surface protein expression was analyzed using the following specific fluorescently conjugated mAbs: CD45 (30-F11), CD11b (M1/70), Ly6C (HK1.4), B220 (RA3-6B2), CD317 (927), F4/80 (CI:A3-1), CD31 (390), and PDGFRβ (APB5). All antibodies were purchased from BioLegend (San Diego, CA).

### Intracellular staining of IFNβ-producing cells

Mice were treated with saline, PDGF-BB, bleomycin, or PDGF-BB + bleomycin, as described above. On the third day, all animals were treated with brefeldin A (FBA) intravenously (500 μg/500 μl saline) [19], prior to a final injection of the respective treatments. After 6 hours, mice were euthanized and 12-mm skin biopsies were harvested and processed into single-cell suspensions as described above. Recovered cells were stained with LIVE/DEAD Fixable Cell Stain Kit (Invitrogen), treated with FcBlock (BD Biosciences, San Jose, CA), and stained with antibodies targeting cell surface markers. Intracellular detection of IFNβ was performed with Intracellular Fixation & Permeabilization Buffer Set (eBioscience, San Diego, CA). Cells were fixed and washed twice with permeabilization buffer and incubated with anti-mouse IFNβ-FITC (rat monoclonal, clone RMMB-1; PBL; 1:25 dilution) at 4°C overnight followed by a final wash. Samples were acquired on a FACS LSR II (Becton Dickinson) and analyzed using FlowJo software (Treestar, Inc.).

### Isolation and analysis of RNA by qPCR

At the indicated time points after treatment, 8-mm lesional skin biopsy specimens were collected and snap-frozen in 2-ml tubes containing 1 ml of TRIzol reagent (Invitrogen, Carlsbad, CA) and 2.3-mm zirconia/silica beads (Biospec, Bartlesville, OK) and subsequently stored at -80°C. Skin biopsies were fully disrupted using a Mini-BeadBeater-16 (Biospec) for 5 minutes in 1-minute intervals and RNA was isolated from the clarified supernatant using Direct-zol RNA MiniPrep columns (Zymo Research, Irvine, CA) according to the manufacturer’s recommendations. One μg total RNA was reverse transcribed using the High-Capacity cDNA Reverse Transcription Kit (Applied Biosystems, Foster City, CA) to generate the cDNA used for quantitative PCR (qPCR) analysis. qPCR analysis was performed using the QuantStudio 12k Flex System (Applied Biosystems).

TaqMan Gene Expression Assay primer and probe sets for all genes, including those for the normalizer GAPDH, were purchased from Applied Biosystems. The relative quantities of mRNA per sample were determined using the ΔΔCt formula as previously described [18]. The following primers were used for qPCR: Mm00434228_m1 (IL1B), Mm00446190_m1 (IL6), Mm00443258_m1 (TNF), Mm00441242_m1 (CCL2), Mm00487796_m1 (MX1), Mm00491265_m1 (RSAD2), Mm00516793_g1 (IRF7), Mm00459183_m1 (IFIH1/MDA1), Mm00545822_m1 (ANGPT2), Mm00516023_m1 (ICAM1), m01178820_m1(TGFB1), and Mm00435546_m1 (PDGFRB).

### Hydroxyproline measurement

Lesional 8-mm punch biopsy skin samples were hydrolyzed with 6N HCl for 18h at 95°C and total collagen content was quantified using QuickZyme Total Collagen Assay (QuickZyme Biosciences) relative to a standard curve according to the manufacturer’s recommendations.

### Statistical analysis

Data were compared using 2-tailed Student’s t tests. Spearman correlation was calculated to measure the degree of association between IBA-1^+^ and CD31^+^ relative area and ulceration area. Logistic regression was fitted for dichotomized ulceration area (ulceration area = 0 and ulceration area > 0) and Receiver operating characteristic (ROC) curve was plotted for the fitted probabilities based on logistic regression to evaluate the predictive accuracy of IBA-1^+^ and CD31^+^ relative area for ulceration. All analyses were performed using R 3.1.0. All data are expressed as mean ± SEM. P values less than 0.05 were considered statistically significant.

## Results

### Exacerbated skin inflammation with evidence of vascular activation in mice treated with PDGF-BB, in combination with bleomycin

**Fig 1:**
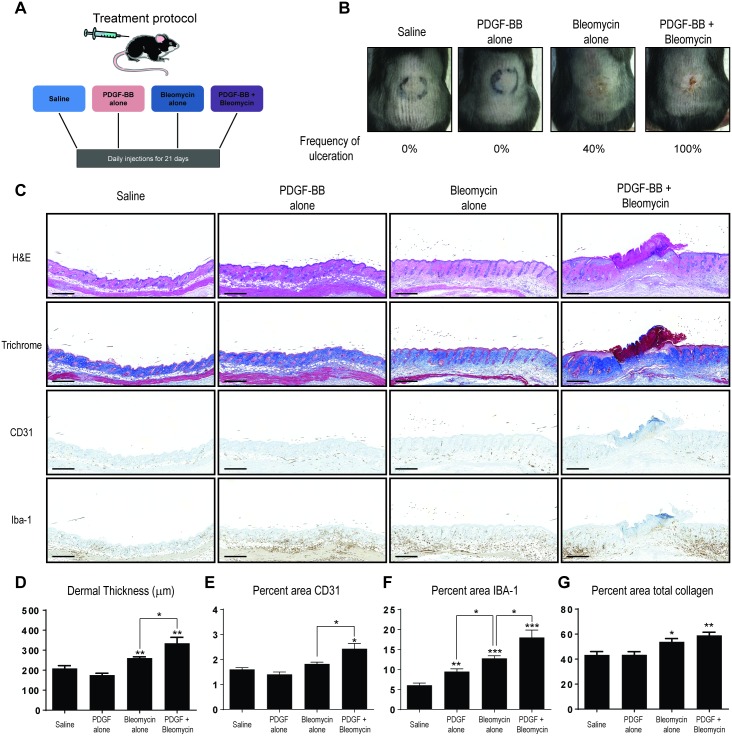
Exacerbated skin inflammation and ulceration in mice treated with combination of PDGF-BB + bleomycin. (A) Outline of experimental design. Intradermal injection of saline, PDGF-BB alone, bleomycin alone, or PDGF-BB + bleomycin were performed daily for 21 consecutive days on female mice at 8 weeks of age. (B) Representative photographs of skin lesions on backs of treated mice. Frequency of ulceration was calculated based on visual examination of H&E stained tissue sections. (C) Representative skin section of treated mice stained by H&E, trichrome, anti-CD31, or anti-IBA-1 immunohistochemistry (scale bar, 500 μm). (D-G) Quantification of dermal thickness (D), percent area CD31 (E) or percent area IBA-1 (F) immunoreactivity, or total collagen (E) as determined by image analysis as described in the methods. Data is representative of at least two experiments with at least 5 mice/group/experiment. *P < 0.05, **P < 0.01, ***P < 0.001, versus saline or between bracketed comparisons shown (student’s *t* test).

**Fig 2:**
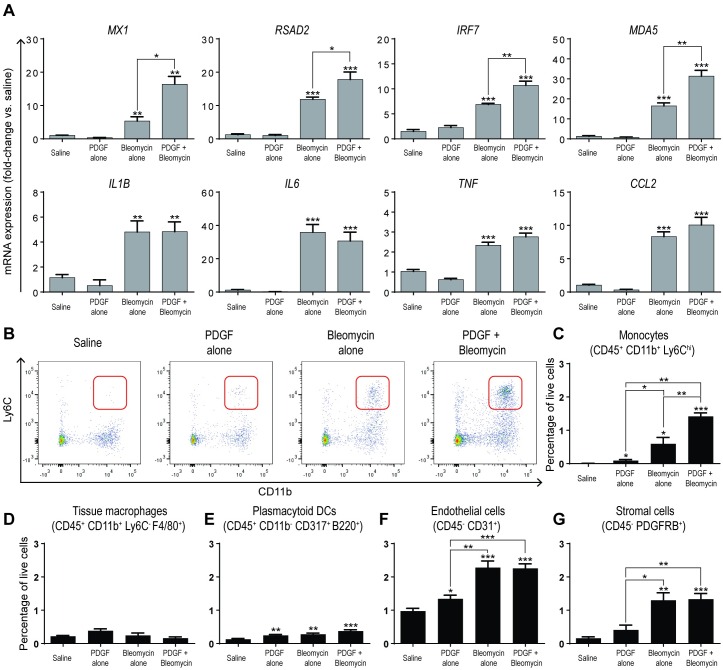
Inflammatory response is skewed towards a Type I IFN-dependent response with increased recruitment of monocytes. (A) Relative mRNA expression levels of the ISGs: *MX1*, *RSAD2*, *IRF7*, or *MDA5* (top) or proinflammatory mediators: *IL1B*, *IL6*, *TNF*, or *CCL2* from lesional skin homogenates of skin biopsies performed by Q-PCR on day 3. (B-G) Single-cell suspensions of skin biopsies obtained from treated mice and analyzed by flow cytometry. (B) Representative dot plot of total CD45-postive leukocytes co-stained with CD11b and Ly6C. (C-G) Proportion of total live cells obtained from each biopsy that stained positive for markers of: (C) monocytes (CD45^+^CD11b^+^Ly6C^hi^), (D) tissue resident macrophages (CD45^+^CD11b^+^F4/80^+^Ly6C^-^), (E) plasmacytoid dendritic cells (CD45^+^CD11b^-^CD317^+^B220^+^), (F) endothelial cells (CD45^-^CD31^+^), or (G) stromal cells (CD45^-^PDGFRβ^+^). Data is representative of at least two experiments with at least 5 mice/group/experiment. *P < 0.05, **P < 0.01, ***P < 0.001, versus saline or between bracketed comparisons shown (student’s *t* test).

In the steady-state, PDGFRβ expression is observed on fibroblasts in the dermis and vascular structures in normal mouse skin (S1 Fig). To determine if PDGFRβ activation in the skin could alter vascular- or immune cell activation in the context of dermal injury and fibrosis, we modified the standard model of intradermal bleomycin injection to include co-administration of PDGF-BB, the ligand for PDGFRβ (Fig 1A). Mice were treated with either saline, bleomycin alone, PDGF-BB alone, or the combination of PDGF-BB + bleomycin, and the resultant dermal injury at day 21 post initiation of treatment was observed. Bleomycin-treated mice had significant dermal inflammation, with ulcerative skin lesions seen in 40% of treated animals (Fig 1B). Consistent with the literature, mice treated with bleomycin alone developed an increase in dermal thickness, monocyte/macrophage cell infiltrates, and total collagen area but showed no evidence of altered vascular density in the skin compared to saline-treated mice (Figs 1C–1G and 2). Mice treated with PDGF-BB alone developed a mild inflammatory reaction with a more modest increase in recruitment of monocyte/macrophage cells to the site of injection, but showed no evidence of ulcerations, dermal thickening, or changes in total collagen as determined by image analysis tissue sections (Fig 1B–1G). In contrast, animals treated with the combination of PDGF-BB + bleomycin exhibited the highest levels of dermal inflammation, with 100% of treated animals developing ulcerative skin lesions (Fig 1B). Further, treatment with single agent PDGF-BB or bleomycin did not affect the density of blood vessels within the tissue, whereas mice treated with PDGF-BB + bleomycin, exhibited a 50% increase percent area of CD31 immunopositivity in the affected tissue compared to those treated with saline (Fig 1E). Mice treated with PDGF-BB + bleomycin also exhibited a greater increase in dermal thickness and monocyte/macrophage cell recruitment when compared to those treated with bleomycin or PDGF-BB alone (Fig 1D–1G). Notably, the combination of PDGF-BB + bleomycin resulted in a 28% and 41% increase in dermal thickness and monocyte/macrophage cell recruitment, respectively, compared to mice treated with single agent bleomycin. In agreement with the image analysis of the Picrosirius Red-stained samples (Fig 1G), quantification of total collagen in lesional skin biopsies using a hydroxyproline assay confirmed that mice treated with the combination of PDGF-BB + bleomycin did not result in a significant increase in total collagen content compared to bleomycin alone (S3 Fig). Thus, no differences were observed in the amount of total collagen in mice treated with the combination of PDGF-BB + bleomycin compared to single agent bleomycin treatment, suggesting that the increased dermal thickness observed PDGF-BB + bleomycin-treated mice is likely due to increased monocyte/macrophage cell recruitment and not fibrosis

To investigate the mechanisms underlying the observed dermal injury, expression analysis of genes related to vascular activation and tissue fibrosis was performed on skin lesion tissue at two different time points: days 3 (early) and 21 (late) post initiation of treatment. Results of the qPCR analysis showed that as early as day 3, the vascular activation markers *ANGPT2* and *ICAM1* and the pro-fibrotic marker *TGFβ*, were all significantly upregulated in mice treated with single agent bleomycin compared to the saline treated mice (S4 Fig). The combination of PDGF-BB + bleomycin resulted in an additional 29.9%, 24.4%, and 28.7% increase in the expression of *ANGPT2*, *ICAM1*, and *TGFB1*, respectively, compared to mice treated with single agent bleomycin. A qualitatively similar gene expression profile was observed in mice on day 21. The expression of *PDGFRB*, the receptor for PDGF-BB, was increased 1.5-fold in mice treated with the combination of PDGF-BB + bleomycin, but not in single agent bleomycin treated mice compared to those treated with saline at day 21.

### Activation of PDGFRβ skews the immune response towards the Type I IFN axis and promotes recruitment of monocytes

In addition to investigating the factors involved in vascular activation and tissue fibrosis in our model, we also analyzed the expression of genes associated with immune cell recruitment and activation in order to assess a potential link between vascular and immune cell activation. For consistency, we chose the day 3 time point for analysis, given that increased expression of vascular activation and tissue fibrosis markers was observed as early as day 3 after initiation of treatment. Gene expression analysis showed that unlike saline or PDGF-BB treated mice, treatment of mice with bleomycin alone consistently induced the expression of ISGs and proinflammatory genes (Fig 2A). Surprisingly, mice treated with PDGF-BB + bleomycin exhibited significantly increased expression of ISGs (> 50% increase vs. bleomycin, on average), but not pro-inflammatory genes, including IL-1β, IL-6, TNF, or CCL2, as compared to bleomycin-treated mice (Fig 2A). These data suggest that combination treatment had skewed the immune response towards the Type I IFN-response.

To further elucidate the cellular changes occurring in response to PDGF-BB + bleomycin treatment, we processed skin biopsies at day 3 post treatment initiation into single-cell suspensions and performed FACS analysis to determine the phenotype of the immune cell infiltrates in the skin lesions at this early time point. We observed the highest level of CD45^+^CD11b^+^Ly6C^hi^ monocyte accumulation in PDGF-BB + bleomycin-treated mice (253.6-fold increase as compared with saline-treated controls), with single agent bleomycin and PDGF-BB-treated mice exhibiting 106.1-fold and 16.1-fold increases, respectively, compared with saline-treated controls (Fig 2B and 2C). The proportion of tissue resident macrophages, defined as CD45^+^CD11b^+^F4/80^+^Ly6C^-^ cells, was not increased in any treatment group relative to saline (Fig 2D), and the proportion of plasmacytoid dendritic cells, which are known to be major producers of Type I IFNs, was increased in all treated groups relative to saline (Fig 2E). The proportion of endothelial cells was increased 1.4-fold in mice treated with PDGF-BB alone and further increased in mice treated with bleomycin or the combination of PDGF-BB + bleomycin (2.3-fold each). The proportion of stromal cells was also increased to 8.2- and 8.4-fold, respectively, in mice treated with bleomycin alone or the combination of PDGF-BB + bleomycin, as compared to saline. Similar results were seen in the absolute number of cells across all comparisons (data not shown). These data show that mice treated with PDGF-BB + bleomycin uniquely exhibited an increased proportion of monocytes. Together, these results confirm that increased ISG expression and monocyte recruitment in response to PDGF-BB + bleomycin treatment is an early distinguishing feature of the combination treatment compared with bleomycin or PDGF-BB single agent treatment.

### Increased accumulation of IFNβ-producing myeloid cells in mice treated with PDGF-BB, in combination with bleomycin

**Fig 3:**
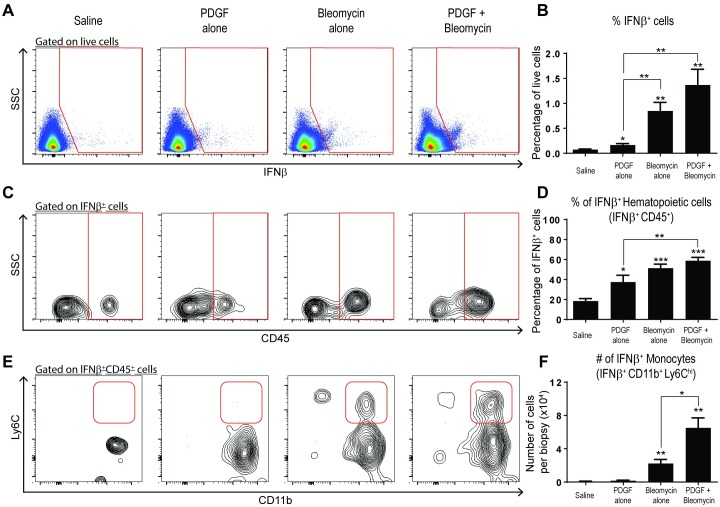
Myeloid cells are a prominent source of IFNβ upon treatment with the combination of PDGF-BB + bleomycin. Wildtype mice were treated with saline, PDGF-BB alone, bleomycin alone, or PDGF-BB + bleomycin were assessed for production of IFNβ by intracellular staining on day 3 post-treatment. (A) Representative flow cytometric plots of intracellular staining of IFNβ^+^ cells from single-cell suspensions obtained from skin biopsies are shown. (B) Proportion of total live cells positive for IFNβ. (C) Representative flow cytometric plots of IFNβ-producing leukocytes (gated IFNβ^+^). (D) Proportion of IFNβ^+^ cells that are leukocytes as defined by expression of CD45. (E) Representative flow cytometric plots of IFNβ-producing monocytes (gated IFNβ^+^CD45^+^). (D) Absolute number of IFNβ-producing monocytes as defined by co-expression of CD11b and Ly6C (gated IFNβ^+^CD45^+^). Data is representative of at least two experiments with at least 5 mice/group/experiment. *P < 0.05, **P < 0.01, ***P < 0.001, versus saline or for bracketed comparisons shown (student’s *t* test).

Since many different cell types can produce Type I IFNs, we sought to identify the cell types that produce IFNβ in the model of dermal PDGF-BB + bleomycin treatment. To accomplish this, we performed intracellular staining to detect the cellular sources of IFNβ at day 3 post-treatment as this was the time point where we observed increased ISG production (Fig 2A). Relative to saline treated mice, mice treated with PDGF-BB alone, bleomycin alone, or PDGF-BB + bleomycin exhibited a 2.2-fold, 11.5 -fold, or 18.6-fold increase, respectively, in percentage of total skin cells producing IFNβ (Fig 3A and 3B). Amongst the IFNβ^+^ cells in saline treated mice, 18% were CD45^+^. However, the proportion of IFNβ^+^ CD45^+^ cells was altered with treatment. Single agent PDGF-BB treatment led to a 2-fold increase in the proportion of CD45^+^ hematopoietic cells that produced IFNβ compared to saline, which was further increased in mice treated with bleomycin alone or PDGF-BB + bleomycin (2.8- and 3.2-fold, respectively). Further analysis revealed that greater than 90% of hematopoietic cells that produced IFNβ were of the myeloid cell lineage and expressed the marker CD11b (Figs 3E and S5). We further quantified the number of infiltrating inflammatory monocytes (CD11b^+^Ly6C^hi^) that produced IFNβ and found an increased proportion of in the PDGF-BB + bleomycin as compared to single agent-treated mice. The highest level, a 99.7-fold increase in the absolute number of IFNβ-producing CD45^+^CD11b^+^Ly6C^hi^ monocytes was found in PDGF-BB + bleomycin-treated mice whereas single-agent bleomycin- and PDGF-BB-treated mice exhibited 33.7-fold and 2.5-fold increases, respectively, compared with saline-treated controls (Fig 3E and 3F). Thus, mice treated with PDGF-BB + bleomycin uniquely exhibited an increased recruitment of IFNβ-producing monocytes.

### IFNAR activation is necessary for PDGF-dependent blood vessel maintenance and monocyte/macrophage cell recruitment

**Fig 4:**
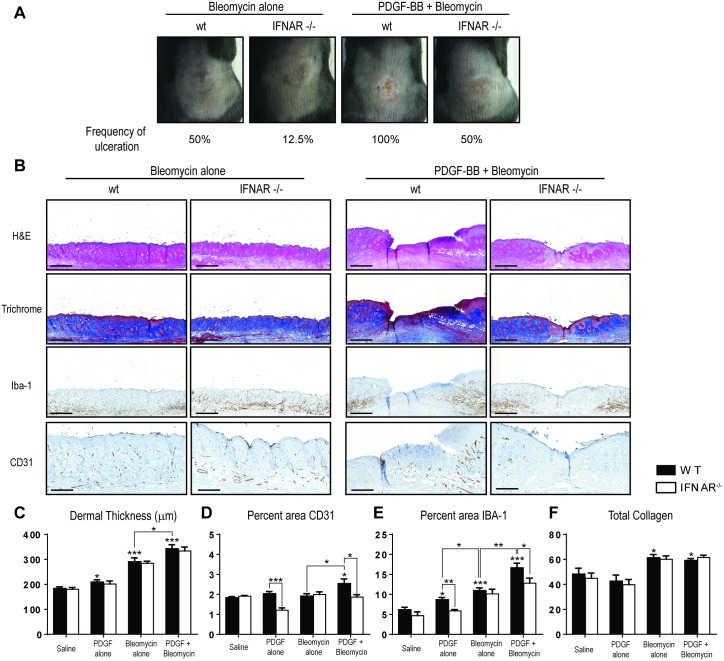
IFNAR activation is necessary for blood vessel stabilization and recruitment of monocyte/macrophage cells in PDGF-BB + bleomycin-treated mice. (A) Representative photographs of skin lesions on backs of treated wildtype or IFNAR-deficient mice. Frequency of ulceration was calculated based on visual examination of H&E stained tissue sections. (B) Representative skin section of treated mice stained by H&E, trichrome, anti-IBA-1, or anti-CD31 immunohistochemistry (scale bar for H&E, trichrome, and anti-IBA-1: 500 μm, scale bar for anti-CD31: 300 μm). (C-F) Quantification of dermal thickness (C), percent area CD31 (E) or percent area IBA-1 (F) immunoreactivity, or total collagen (D) staining as determined by image analysis as described in the methods. *P < 0.05, **P < 0.01, ***P < 0.001, versus saline or for bracketed comparisons shown (student’s *t* test).

Since increased production of ISGs and recruitment of IFNβ-producing monocytes were distinguishing features in PDGF-BB + bleomycin-treated mice compared with those treated with single agent bleomycin, we hypothesized that IFNAR signaling may drive exacerbation of dermal injury in these mice. To test this hypothesis, we compared the skin pathology observed in wildtype vs. IFNAR-deficient mice treated with single agent or the combination of PDGF-BB + bleomycin. Wildtype mice treated with bleomycin or PDGF-BB + bleomycin developed skin lesions with high frequencies of ulceration (50% and 100%, respectively) (Fig 4A), similar to the results shown in Fig 1B. In contrast, mice deficient in IFNAR exhibited a reduced frequency of ulceration with only 12.5% and 50% of mice treated with bleomycin or PDGF-BB + bleomycin developing lesions, respectively (Fig 4A).

The lack of IFNAR signaling did not affect the dermal thickening or total collagen observed in mice treated with single agent or PDGF-BB + bleomycin (Figs 4B, 4C, 4F and S6). However, in contrast to the significant increase in blood vessel density seen in wildtype mice treated with PDGF-BB + bleomycin as compared to controls, there was no increase in blood vessel density observed in IFNAR-deficient mice treated with the combination of PDGF-BB + bleomycin (Fig 4B and 4D). Surprisingly, IFNAR-deficient mice, but not wildtype mice, treated with PDGF-BB alone also exhibited a reduction in blood vessel density, suggesting that IFNAR-signaling may have an important role in blood vessel stabilization after PDGFRβ activation. The dependence of PDGF-BB-induced mechanisms on IFNAR signaling also extended to the accumulation of IBA-1^+^ monocyte/macrophage cells (Fig 4B and 4E). The increased recruitment of monocyte/macrophage cells in PDGF-treated animals was completely dependent on IFNAR signaling as IBA-1-positivity was reduced in IFNAR-deficient mice to levels observed in saline-treated animals. Similarly, mice treated with the combination of PDGF-BB + bleomycin exhibited a reduction in IBA-1-accumulation in IFNAR deficient mice to levels observed in animals treated with bleomycin alone. In contrast, the accumulation of monocyte/macrophage cells in mice treated with bleomycin alone was not dependent on IFNAR signaling. Importantly, there was a significant positive correlation between the extent of tissue ulceration and the magnitude of monocyte/macrophage cell infiltration (as assessed by IBA-1^+^ relative area) and blood vessel density (as assessed by CD31^+^ relative area) (S7A and S7B Fig). Further, ROC curve analysis was applied to evaluate the predictive accuracy of monocyte/macrophage cell infiltration and blood vessel density for presence or absence of ulceration. ROC analysis revealed an area under curve (AUC) of 0.86 and 0.73 for IBA-1^+^ and CD31^+^ relative area, respectively, in predicting ulceration. The combination of IBA-1^+^ and CD31^+^ relative area did not improve the predictive accuracy for ulceration compared to IBA-1 alone (p > 0.05). Thus, the extent of monocyte/macrophage cell infiltration is a better predictor of ulceration than blood vessel density. Lastly, the production of the ISGs *MX1*, *RSAD2*, *IRF7*, and *MDA5* was completely abrogated in IFNAR deficient mice at both early (day 3) and late (day 21) time points in mice treated with bleomycin alone or PDGF-BB + bleomycin (S8 Fig). Taken together, these results demonstrate that PDGF-BB-dependent blood vessel maintenance and monocyte/macrophage cell recruitment require IFNAR activation.

## Discussion

Vasculopathy and aberrant immune cell activation are early events in SSc that precede tissue fibrosis, which occurs later on as the disease progresses. In the early stages of SSc, dysregulated production of pro-angiogenic and inflammatory factors has been observed, but a mechanistic link between these two processes has not been identified. Here, we provide evidence that chronic activation of PDGFRβ in a novel model of skin fibrosis promotes vascular activation and skews the system towards a Type I IFN-driven response. PDGFRβ activation was found to significantly increase the number of inflammatory monocytes recruited to the tissue. These monocytes were a significant source of IFNβ at the site of disease. The addition of PDGFRβ in this model resulted in increased inflammation, dermal thickening, angiogenesis, and exacerbated tissue injury. Interestingly, PDGF-BB-dependent functions of tissue injury, blood vessel maintenance, and monocyte/macrophage cell recruitment in the skin were found to be dependent on IFNAR signaling. Taken together, these data suggest a novel link between PDGFRβ-driven vascular activation and Type I IFN-dependent vascular density, monocyte/macrophage cell recruitment and tissue damage.

**Fig 5:**
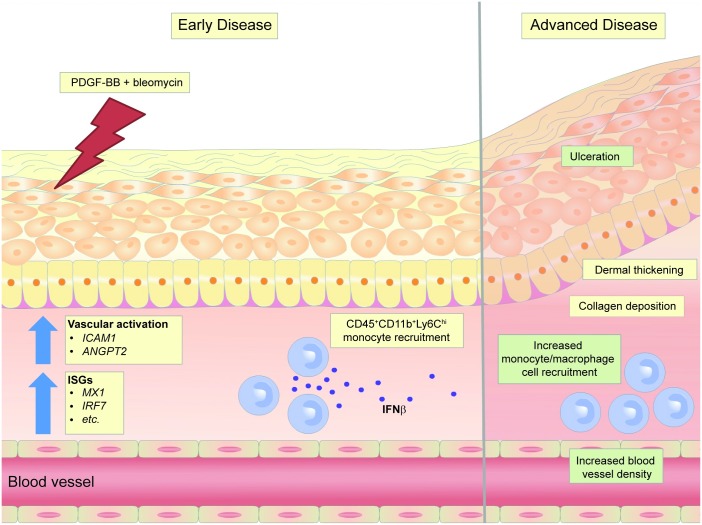
Model of chronic activation of PDGFRβ in a model of skin fibrosis. Chronic activation of PDGFRβ in a model of skin fibrosis promotes vascular activation (*ICAM1* and *ANGPT2*) and Type I IFN-dependent inflammatory response (ISGs). PDGFRβ activation significantly increases the numbers of IFNβ-producing inflammatory monocytes recruited to the tissue. PDGF-BB-dependent functions of tissue injury, blood vessel maintenance, and monocyte/macrophage cell recruitment in the skin were found to be dependent on IFNAR signaling (highlighted in green) in this model of dermal fibrosis.

The PDGFRβ axis is implicated in a number of fibrotic diseases. Given the key role of PDGF-BB as a mitogenic factor in mesenchymal cells, many prior studies have focused on its role in the expansion of collagen-producing myofibroblasts [20]. However, in SSc, aberrant activation of PDGFRβ is most prominent in the perivascular region of affected skin and not in the surrounding fibroblasts [21], yet the significance of this distinction has been underexplored. In our efforts to dissect the mechanisms whereby PDGFRβ activation contributes to disease progression, we have found that exogenous intradermal administration of PDGF-BB in combination with bleomycin activates the vasculature, as shown by increased CD31 density, and ANGPT2 and ICAM1 gene expression. This activation state was associated with increased recruitment of monocytes to the dermis, ultimately leading to tissue ulceration, likely due to excessive inflammation. We hypothesize that PDGFRβ activation in perivascular cells resulted in crosstalk with endothelial cells, leading to proinflammatory changes including ICAM1 upregulation and subsequently, increased inflammatory monocyte infiltration. Surprisingly, PDGFRβ activation skewed the bleomycin-induced immune response to a Type I IFN phenotype, which was necessary for the increased CD31 density, monocyte recruitment, and tissue ulceration (Fig 5). Consistent with this observation, we also found that IFNAR signaling was required to maintain PDGF-BB-dependent vascular stability and monocyte recruitment in the absence of bleomycin challenge. Interestingly, the overall impact of PDGF-BB administration on dermal fibrosis was nominal, and although we observed an increased expression of TGFB1 in mice treated with PDGF-BB + bleomycin, total collagen accumulation was not significantly affected compared to mice treated with bleomycin alone. Taken together, these findings suggest a novel mechanism to explain the functional outcome of aberrant PDGFRβ activation in perivascular cells in SSc, by demonstrating PDGF-BB-induced skewing to a Type I IFN-driven immune response. While the Type I IFN axis has been previously implicated in SSc in genome-wide association studies and reports of the presence of an interferon signature in patients [22], our study showing the IFNAR-dependence of a more severe skin phenotype further supports a potential role of Type I IFNs as a driver of dermal disease in SSc.

The contradiction between insufficient angiogenesis and the simultaneous overexpression of pro-angiogenic factors, such as VEGF and PDGF, in the lesions of these patients has been a key conundrum in the pathogenesis of SSc. With regards to VEGF, studies using VEGF-transgenic mice have demonstrated that overexpression of VEGF on one allele can support angiogenesis whereas bi-allelic expression of the transgene cannot, demonstrating that the magnitude of VEGF production can influence the stability of blood vessels [23]. While we did not observe a loss in dermal blood vessels in mice treated with PDGF-BB alone, yet an increase in blood vessel density in mice treated with the combination of PDGF-BB + bleomycin was apparent. This may be due to the level of PDGF-BB dosing or the time-course of PDGFRβ–activation in our murine model. Nevertheless, our results identify a role of Type I interferons in the regulation of PDGF-dependent blood vessel maintenance. Loss of IFNAR-signaling led to a significant reduction in blood vessels in both mice treated with PDGF-BB single agent and the combination of PDGF-BB + bleomycin. Since PDGFRβ expression was ubiquitous in various cell types found in normal mouse skin, it is unclear if the observed effects of PDGF-BB on dermal vascular density is mediated by PDGFRβ activation on perivascular cells, or other PDGFRβ-expressing cells, such as fibroblasts. Further investigation is warranted to explore these aspects.

It has been widely thought that the microvascular changes in SSc could be the result of aberrant production of pro-angiogenic factors [24]. However, recently it was shown that patients with gain-of-function mutations in STING, a cytosolic DNA sensor, leading to constitutive expression of IFNβ, present with microvascular abnormalities similar to those observed in the nail-fold capillaries of SSc patients, namely nail-fold capillary tortuosity, capillary-loop loss, telangiectasia, and digital ulcers [15]. Type I IFN dysregulation is also associated with the development of the cerebrovascular disease, Aicardi-Goutières syndrome [25]. Further supporting the role of IFNs in SSc, the development of nail-fold capillary tortuosity, telangiectasia, and digital ulcers have been reported in patients being treated with IFNs for viral infections [13,26]. Our observation that PDGF-BB-dependent contributions to blood vessel maintenance require IFNAR signaling provides evidence linking PDGF-BB-dependent activation of the Type I IFN axis and altered vascular homeostasis, and suggests a novel mechanism for the vasculopathy seen in early stage SSc. However, it should be noted that in human fibroblasts, poly I:C-induced Type I IFN production was shown to inhibit pro-fibrotic responses, suggesting that Type I IFN production in SSc may also have a protective role in limiting fibrosis [27]. Further investigation will be necessary to determine the exact mechanisms whereby aberrant type I IFN production contributes to SSc pathogenesis.

Tyrosine kinase inhibitors (TKIs) that can block TGFRβ and PDGFRβ- pathways have been evaluated in patients with SSc. One such TKI, nilotinib, has been evaluated in a Phase IIa open-label study in patients with early, diffuse SSc. A subset of patients receiving nilotinib was found to exhibit a marked reduction in modified Rodnan Skin Score (MRSS), a measure of skin thickening, which correlated with a significant reduction in the expression of inflammatory genes in the skin, including ISGs [28]. Since nilotinib can affect a number of receptor tyrosine kinases, it is not immediately clear whether the decrease in interferon signaling observed in nilotinib-treated patients is a result of PDGFRβ inhibition or whether other receptors are involved. However, our current study provides support for the notion that blocking PDGFRβ signaling would decrease interferon signaling.

The lack of murine models that recapitulate the distinct clinical features of SSc has made it difficult to develop effective therapies to treat patients. The bleomycin-induced dermal fibrosis model is the most commonly used preclinical model of SSc [29]. This model recapitulates many of the inflammatory changes in early stage SSc, such as the production of reactive oxygen species, and autoantibody production. However, evidence of vasculopathy is poorly represented in this model [30]. In addition, the vascular and inflammatory aspects observed in the bleomycin model incompletely model the full spectrum of pathologies observed in SSc patients. Owing to this, many of the anti-inflammatory therapies that have shown clinical promise in the bleomycin model were later found to be ineffective in SSc patients in the clinic [30]. Here, we have modified the bleomycin model to include PDGFRβ activation, resulting in a novel model that may better recapitulate the subset of human SSc patients with an increased interferon signature. Using this model, we have identified a novel role for PDGF-BB dependent vascular function and immune cell recruitment, both of which depend on the activation of IFNAR. These results provide critical insight into the early vascular and immune-mediated pathologies associated with SSc. In the future, this novel model should be useful in the development of novel therapies for SSc, where there is currently high unmet need for disease modifying therapies.

## Supporting Information

S1 Fig PDGFRβ expression in normal murine skin. Representative skin section of normal mice stained with anti-PDGFRβ (left) or co-stained with anti-PDGFRβ and anti-CD31 (right).
(TIF)

S2 Fig Higher magnification images wildtype mice treated with PDGF-BB + bleomycin. Representative skin section of treated mice stained by H&E, trichrome, anti-CD31, or anti-IBA-1 immunohistochemistry (scale bar, 300 μm).
(TIF)

S3 Fig Quantification of total collagen. Lesional 8-mm punch biopsy skin samples were harvested at day 21 of treatment regimen and assessed for total collagen using a hydroxyproline assay. **P < 0.01, versus saline (student’s *t* test).
(TIF)

S4 Fig Increased expression of vascular activation- and tissue fibrosis-related genes. Relative mRNA expression levels of *ANGPT2*, *ICAM1*, *TGFB1*, or *PDGFRB* from lesional skin homogenates of skin biopsies performed by Q-PCR on days 3 (top) or day 21 (bottom) of treatment regimen. Data is representative of at least two experiments with at least 5 mice/group/experiment. *P < 0.05, **P < 0.01, ***P < 0.001, versus saline or for bracketed comparisons shown (student’s *t* test).
(TIF)

S5 Fig Myeloid cells are the predominant leukocyte population that produces IFNβ. (A) Proportion of CD45^+^IFNβ^+^ cells that are myeloid cells as defined by expression of CD11b. (B) Absolute number of IFNβ-producing myeloid cells as defined by expression of CD11b (gated IFNβ^+^CD45^+^). Data is representative of at least two experiments with at least 5 mice/group/experiment. *P < 0.05, **P < 0.01, ***P < 0.001, versus saline or for bracketed comparisons shown (student’s *t* test).
(TIF)

S6 Fig Higher magnification images wildtype and IFNAR-deficient mice treated with PDGF-BB + bleomycin. Representative skin section of treated mice stained by H&E, trichrome, or anti-IBA-1 immunohistochemistry (scale bar, 300 μm).
(TIF)

S7 Fig Spearman rank correlation and ROC analysis of monocyte/macrophage cell recruitment and vessel density with ulceration area. (A-B) A scatter plot of all treatment groups that were analyzed by spearman rank correlation is shown for IBA-1 (A) and CD31 (B) percent area immunoreactivity compared to area of ulceration (r_s_ = 0.56 for IBA-1 and r_s_ = 0.33 for CD31). (C) The logistic regression and ROC curve analyses were applied to evaluate the predictive accuracy of IBA-1, CD31, or the combination of IBA-1 and CD31 percent area immunoreactivity for presence or absence of ulceration. *P*-values were calculated relative to the null hypothesis for IBA-1 and CD31 or relative to IBA-1 alone for the combination of IBA-1 and CD31 percent area. N = 8 samples per group, two independent studies, 104 total data points. *P-value relative to IBA-1 percent area alone.
(TIF)

S8 Fig Expression of ISGs in lesional skin biopsies obtained from wildtype and IFNAR-deficient mice. Relative mRNA expression levels of *MX1*, *RSAD2*, *IRF7*, or *MDA5* from lesional skin homogenates of skin biopsies performed by Q-PCR on days 3 (top) or day 21 (bottom) of treatment regimen. *P < 0.05, **P < 0.01, ***P < 0.001, versus saline or for bracketed comparisons shown (student’s *t* test).
(TIF)
