# Supplementary figures and images for: PDGF-BB Promotes Type I IFN-Dependent Vascular Alterations and Monocyte Recruitment in a Model of Dermal Fibrosis
Source: PLoS One. 2016 Sep 12;11(9):e0162758. doi: 10.1371/journal.pone.0162758 (PMC5019454; doi:10.1371/journal.pone.0162758)

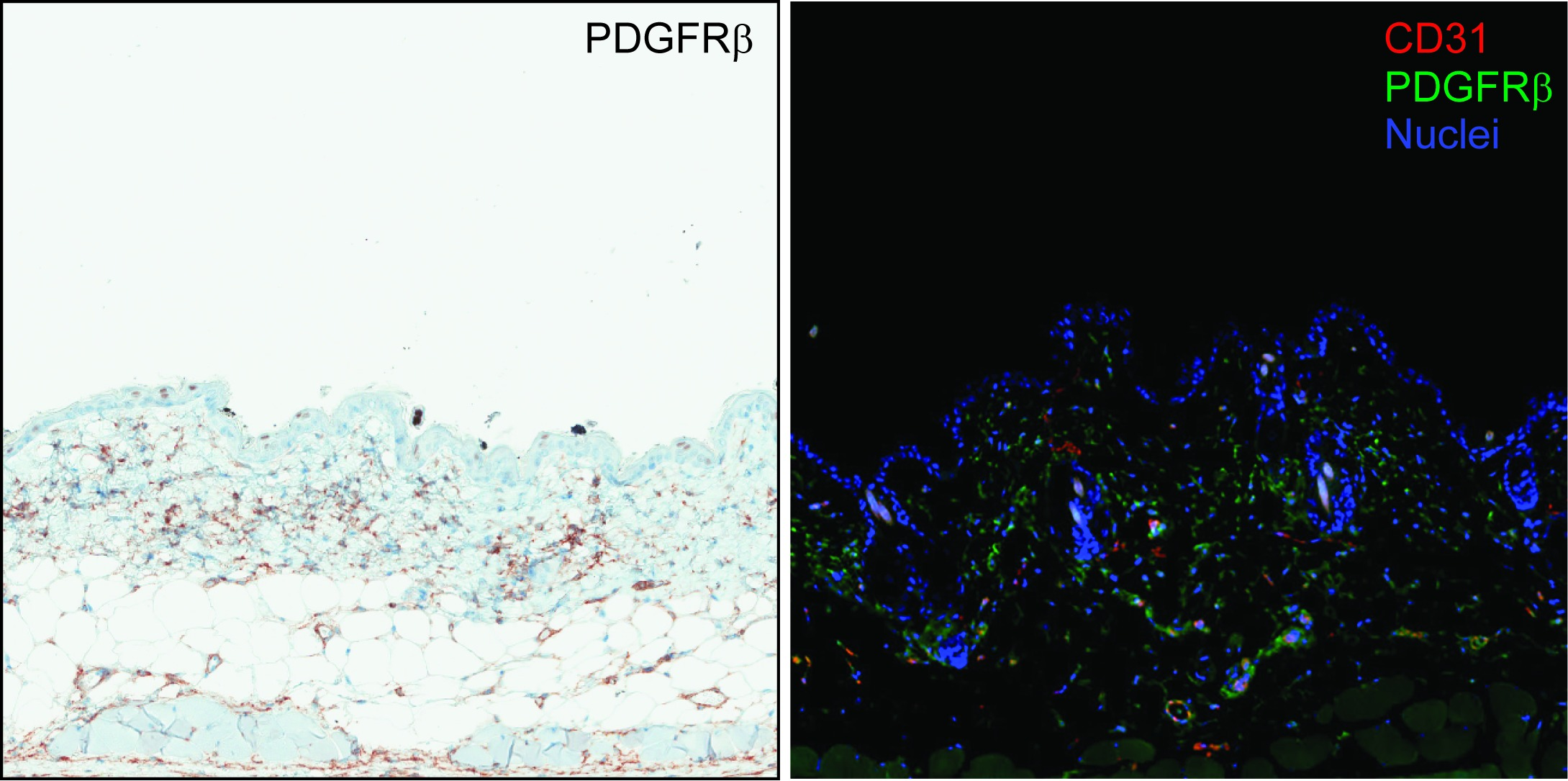

Supplement: S1 Fig — Representative skin section of normal mice stained with anti-PDGFRβ (left) or co-stained with anti-PDGFRβ and anti-CD31 (right). (TIF) [file pone.0162758.s001.tif]

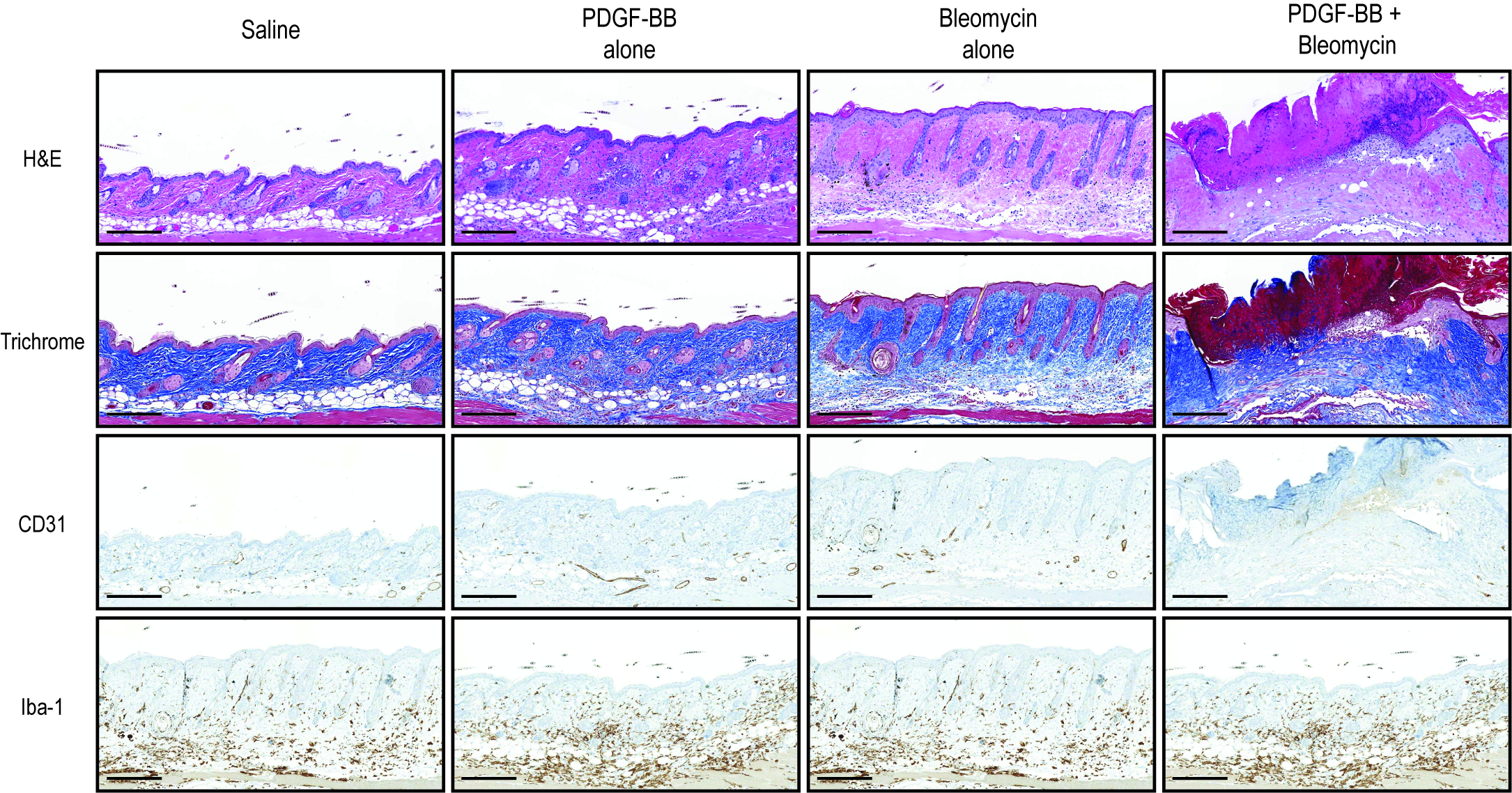

Supplement: S2 Fig — Representative skin section of treated mice stained by H&E, trichrome, anti-CD31, or anti-IBA-1 immunohistochemistry (scale bar, 300 μm). (TIF) [file pone.0162758.s002.tif]

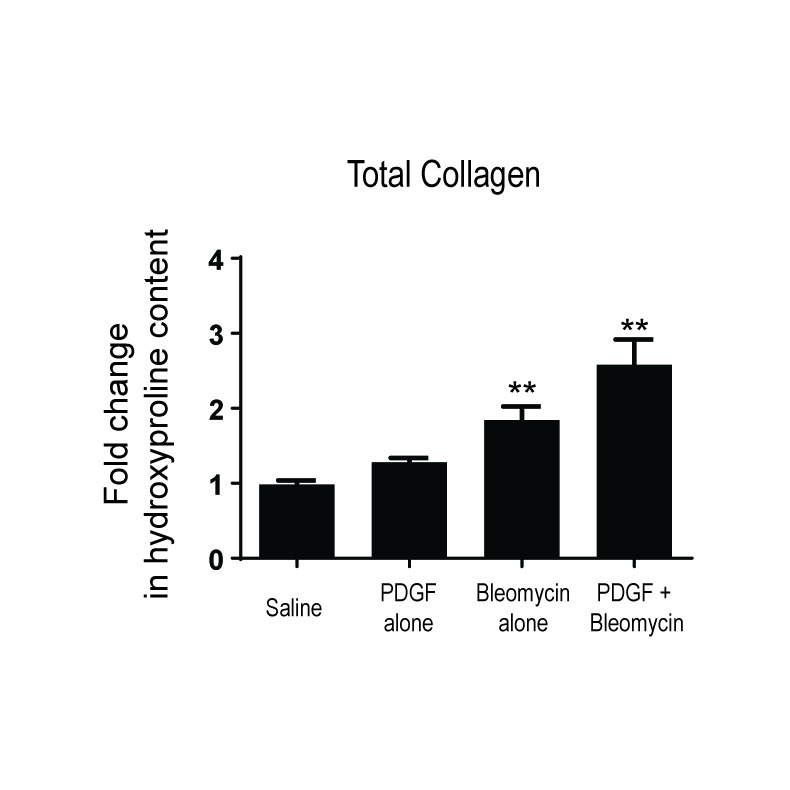

Supplement: S3 Fig — Lesional 8-mm punch biopsy skin samples were harvested at day 21 of treatment regimen and assessed for total collagen using a hydroxyproline assay. **P < 0.01, versus saline (student’s t test). (TIF) [file pone.0162758.s003.tif]

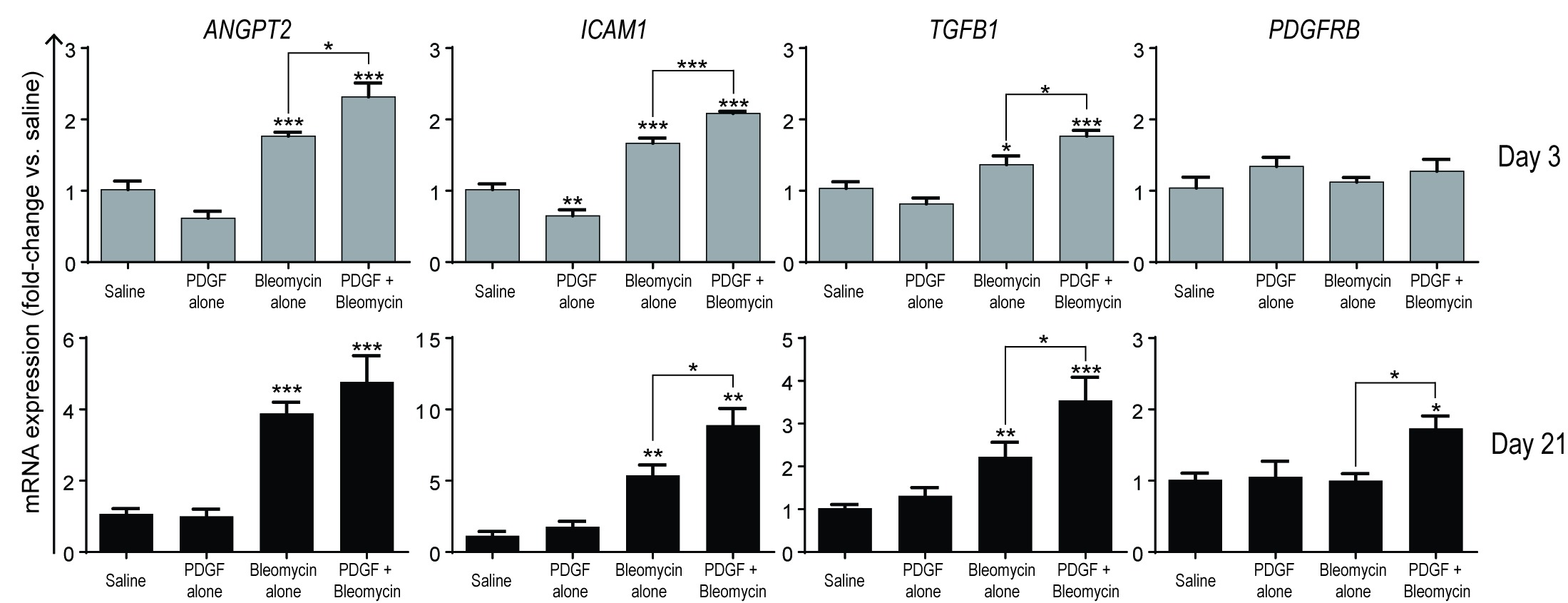

Supplement: S4 Fig — Relative mRNA expression levels of ANGPT2, ICAM1, TGFB1, or PDGFRB from lesional skin homogenates of skin biopsies performed by Q-PCR on days 3 (top) or day 21 (bottom) of treatment regimen. Data is representative of at least two experiments with at least 5 mice/group/experiment. *P < 0.05, **P < 0.01, ***P < 0.001, versus saline or for bracketed comparisons shown (student’s t test). (TIF) [file pone.0162758.s004.tif]

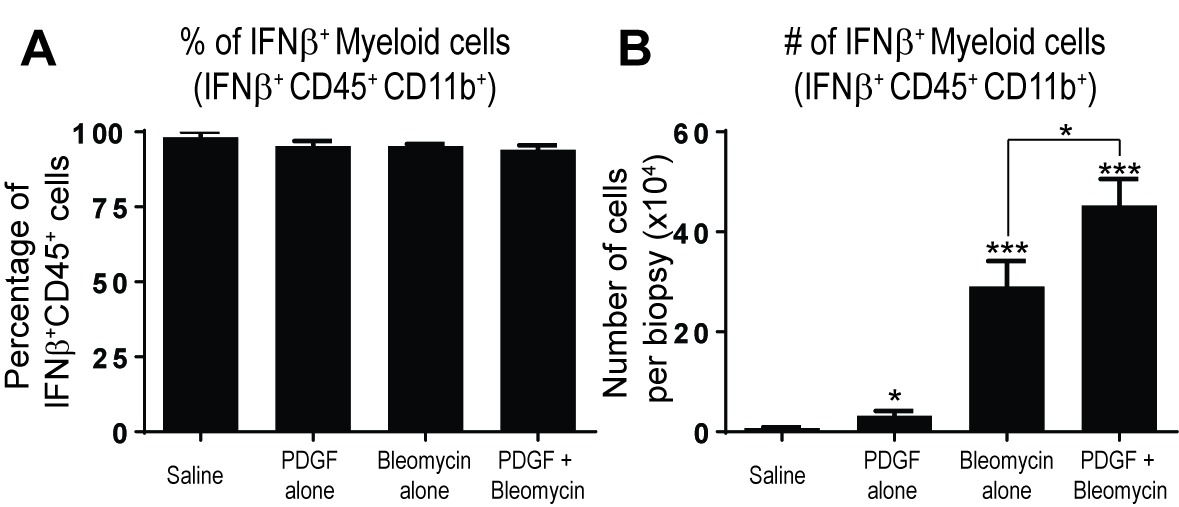

Supplement: S5 Fig — (A) Proportion of CD45+IFNβ+ cells that are myeloid cells as defined by expression of CD11b. (B) Absolute number of IFNβ-producing myeloid cells as defined by expression of CD11b (gated IFNβ+CD45+). Data is representative of at least two experiments with at least 5 mice/group/experiment. *P < 0.05, **P < 0.01, ***P < 0.001, versus saline or for bracketed comparisons shown (student’s t test). (TIF) [file pone.0162758.s005.tif]

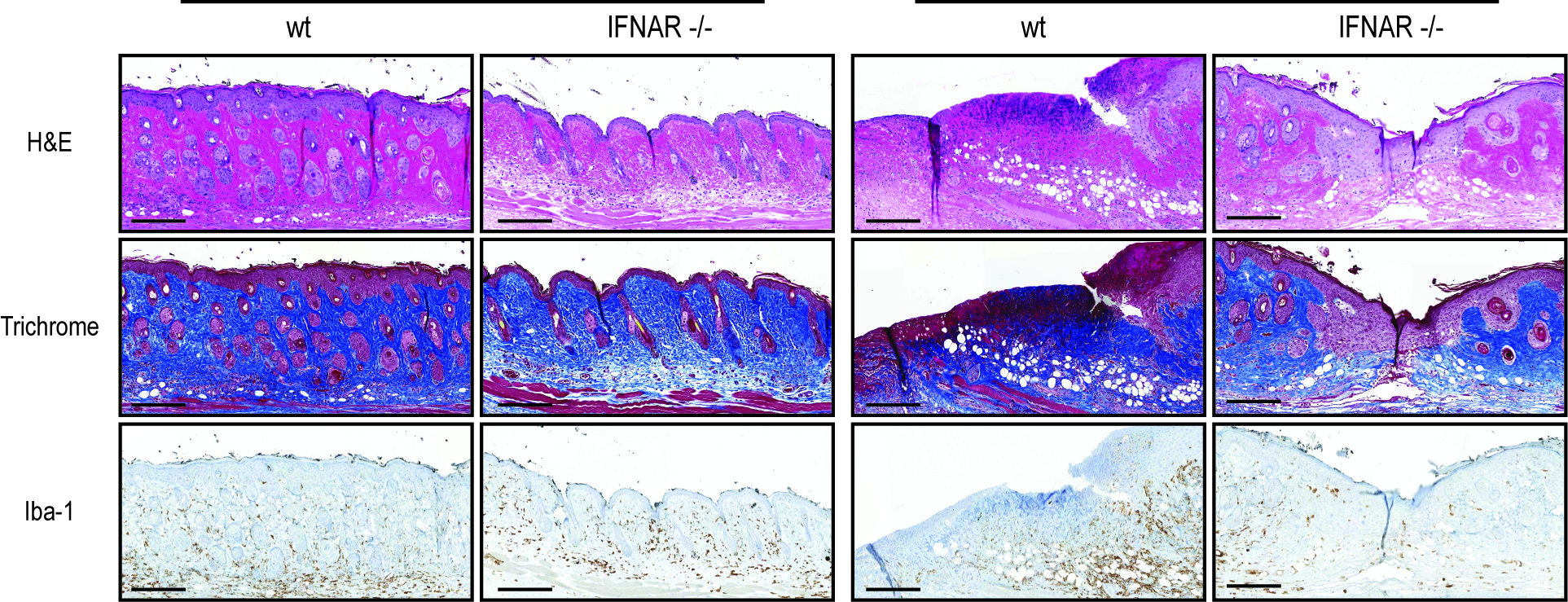

Supplement: S6 Fig — Representative skin section of treated mice stained by H&E, trichrome, or anti-IBA-1 immunohistochemistry (scale bar, 300 μm). (TIF) [file pone.0162758.s006.tif]

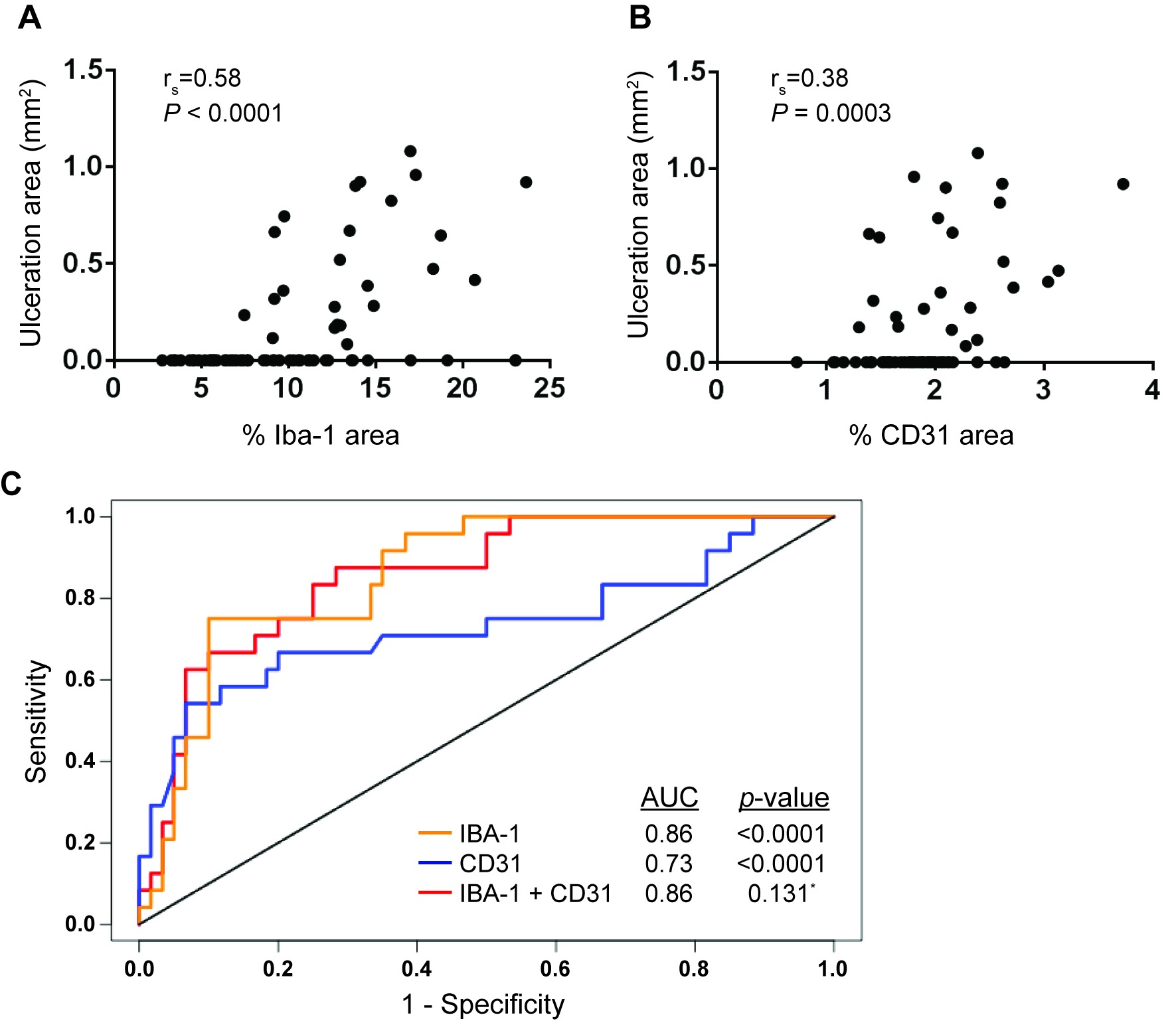

Supplement: S7 Fig — (A-B) A scatter plot of all treatment groups that were analyzed by spearman rank correlation is shown for IBA-1 (A) and CD31 (B) percent area immunoreactivity compared to area of ulceration (rs = 0.56 for IBA-1 and rs = 0.33 for CD31). (C) The logistic regression and ROC curve analyses were applied to evaluate the predictive accuracy of IBA-1, CD31, or the combination of IBA-1 and CD31 percent area immunoreactivity for presence or absence of ulceration. P-values were calculated relative to the null hypothesis for IBA-1 and CD31 or relative to IBA-1 alone for the combination of IBA-1 and CD31 percent area. N = 8 samples per group, two independent studies, 104 total data points. *P-value relative to IBA-1 percent area alone. (TIF) [file pone.0162758.s007.tif]

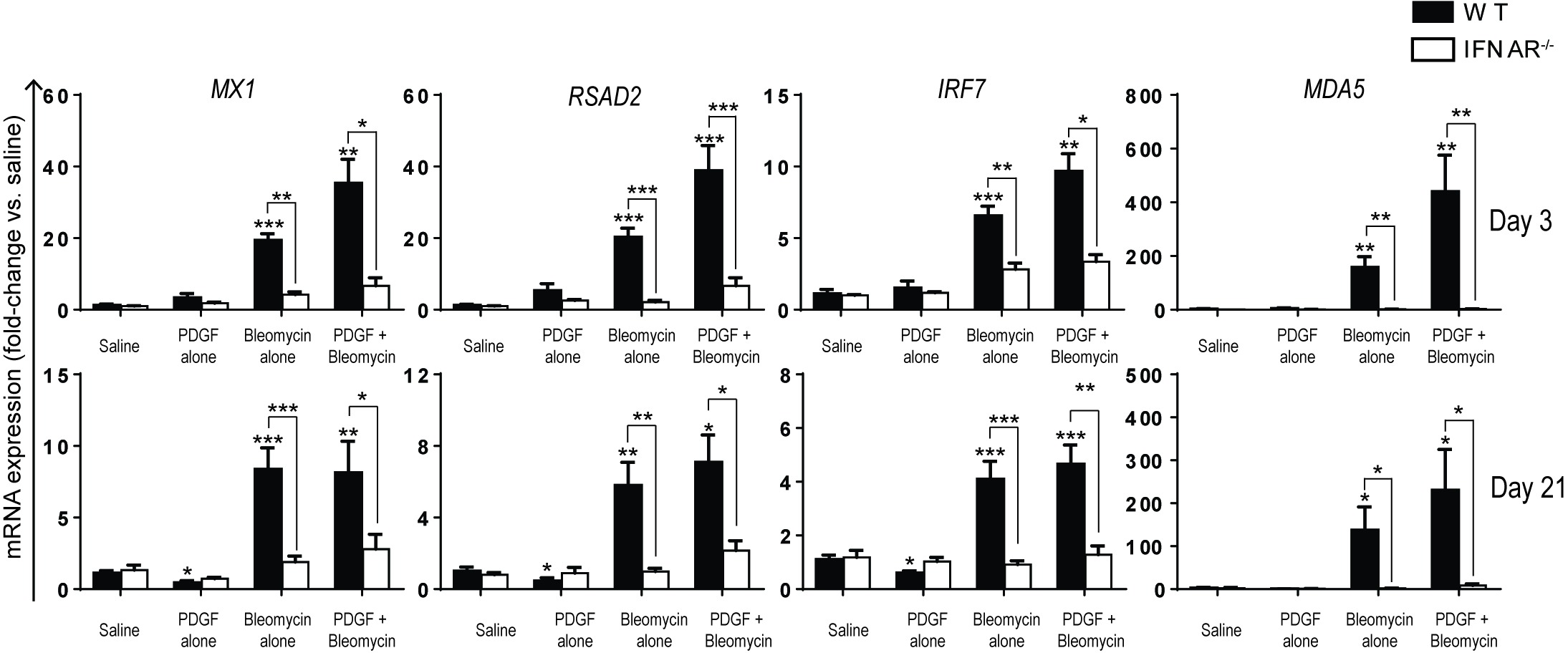

Supplement: S8 Fig — Relative mRNA expression levels of MX1, RSAD2, IRF7, or MDA5 from lesional skin homogenates of skin biopsies performed by Q-PCR on days 3 (top) or day 21 (bottom) of treatment regimen. *P < 0.05, **P < 0.01, ***P < 0.001, versus saline or for bracketed comparisons shown (student’s t test). (TIF) [file pone.0162758.s008.tif]
